# Complete Genome Sequences of the Three African Horse Sickness Virus Strains from a Commercial Trivalent Live Attenuated Vaccine

**DOI:** 10.1128/genomeA.00814-15

**Published:** 2015-08-20

**Authors:** Alan J. Guthrie, Peter Coetzee, Darren P. Martin, Carina W. Lourens, Estelle H. Venter, Camilla T. Weyer, Christopher Joone, Misha le Grange, Cindy K. Harper, Peter G. Howell, N. James MacLachlan

**Affiliations:** a Equine Research Centre, Faculty of Veterinary Science, University of Pretoria, Onderstepoort, South Africa; b Department of Veterinary Tropical Diseases, Faculty of Veterinary Science, University of Pretoria, Onderstepoort, South Africa; c Department of Integrative Biomedical Sciences, Computational Biology Group, Institute of Infectious Disease and Molecular Medicine, University of Cape Town, Rosebank, Cape Town, South Africa; d Veterinary Genetics Laboratory, Faculty of Veterinary Science, University of Pretoria, Onderstepoort, South Africa; e Department of Pathology, Equine Viral Disease Laboratory, Microbiology and Immunology, School of Veterinary Medicine, University of California, Davis, California, USA

## Abstract

This is a report of the complete genome sequences of plaque-selected isolates of each of the three virus strains included in a South African commercial trivalent African horse sickness attenuated live virus vaccine.

## GENOME ANNOUNCEMENT

African horse sickness (AHS) is an arthropod-borne disease of equids that is caused by African horse sickness virus (AHSV) (genus *Orbivirus*, family *Reoviridae*). The genome of AHSV is composed of 10 segments of double-stranded RNA (dsRNA) collectively encoding seven structural proteins and four nonstructural proteins (1). In South Africa, a polyvalent AHS attenuated live virus (ALV) vaccine is manufactured by Onderstepoort Biological Products (OBP) Ltd. This vaccine is supplied in two separate vials, each of which contains different AHSV serotypes; bottle I is trivalent and contains serotypes 1, 3, and 4, while bottle II is tetravalent and contains serotypes 2, 6, 7, and 8 (2).

Here, we report the full-genome sequences of AHSV-Labstr/ZAF/1998/OBP-116 serotype 1, AHSV-Labstr/ZAF/1998/OBP-116 serotype 3, and AHSV-Labstr/ZAF/1998/OBP-116 serotype 4, which were isolated from bottle I of the AHS-ALV vaccine (batch 116; Onderstepoort Biological Products [OBP] Ltd., Onderstepoort, South Africa). The individual serotypes were independently isolated using plaque selection on Vero cells in the presence of heterologous antibody to the other serotypes contained in the trivalent vaccine, as previously described for bluetongue virus (3). Each of these viruses was then passaged between one and three times on monolayers of baby hamster kidney (BHK-21) cells grown in 25-cm^2^ tissue culture flasks. AHSV dsRNA was extracted from virus-infected cells using TRIzol reagent (Life Technologies, Johannesburg, South Africa). Sequencing templates were prepared by using sequence-independent whole-genome reverse transcription-PCR (RT-PCR) ampliﬁcation (4). PCR amplicons were sequenced on an Illumina MiSeq sequencer (Inqaba Biotechnical Industries [Pty] Ltd., Pretoria, South Africa) using the Nextera XT DNA sample preparation kit and 300-bp paired-end V3 Illumina chemistry. Illumina sequence reads were analyzed using Geneious 8.1.5. A combination of *de novo* assembly followed by mapping was used to obtain the full-length genome sequences of the three viruses.

The full-genome sequences of the attenuated AHSV serotype 1 (HS29/62-attenuated) and serotype 4 (HS32/62-attenuated) viruses, which are precursors of the respective AHS-ALV virus strains, are available from GenBank under the accession numbers FJ183364 to FJ183373 and KM820849 to KM820858, respectively. The pairwise nucleotide sequence identity of AHSV-Labstr/ZAF/1998/OBP-116 serotype 1 and HS29/62-attenuated was 99.643%, while that of AHSV-Labstr/ZAF/1998/OBP-116 serotype 4 and HS32/62-attenuated was 99.625%.

The full-genome sequences of AHSV serotype 3 (HS13/63), from which AHSV-Labstr/ZAF/1998/OBP-116 serotype 3 was derived, are available from GenBank under the accession numbers KM886354 to KM886363. The pairwise nucleotide sequence identity between AHSV-Labstr/ZAF/1998/OBP-116 serotype 3 and HS13/63 over nine of their 10 genome segments was >99.7%, while that for the segment carrying the gene encoding the highly conserved VP1 protein was appreciably lower, at 94.86%. Further analysis of these sequences using RDP4.42 (5) (with default settings, except that we invoked the “scan for reassortment and recombination” setting) clearly showed that AHSV-Labstr/ZAF/1998/OBP-116 serotype 3 is a reassortant (*P* < 2.196 × 10^-21^ for all seven of the different recombination/reassortment detection methods implemented in RDP4) composed of nine genome segments likely derived from an AHSV serotype 3 ancestor closely related to HS13/63 (accession numbers KM886355 to KM886363), and the segment encoding VP1 likely derived from an AHSV serotype 1 ancestor closely related to HS29/62-ALV (accession no. FJ183364).

### Nucleotide sequence accession numbers.

The AHSV-Labstr/ZAF/1998/OBP-116 serotype 1, AHSV-Labstr/ZAF/1998/OBP-116 serotype 3, and AHSV-Labstr/ZAF/1998/OBP-116 serotype 4 sequences have been deposited in GenBank under the accession numbers KT030330 to KT030339, KT030340 to KT030349, and KT030350 to KT030359, respectively.
